# The potential of in-ovo probiotics to replace antibiotic growth promoters in broiler diets: Effect on performance, meat quality, cecal fermentation and oxidative stress indicators

**DOI:** 10.1016/j.psj.2026.107026

**Published:** 2026-04-28

**Authors:** Victor Grospiron, Jean-Michel Allard Prus, Antony T. Vincent, Nabeel Alnahhas

**Affiliations:** a Department of Animal Science, Faculty of Agricultural and Food Sciences, Université Laval, Quebec City, Quebec G1V 0A6, Canada; b Scott Hatchery, Scott, Quebec G0S 3G0, Canada; c Swine and Poultry Infectious Diseases Research Center, Université de Montréal, Saint-Hyacinthe, Quebec G1V 4G5, Canada

**Keywords:** Broiler chickens, In-ovo probiotics, Antibiotic growth promoter, Growth, Meat quality

## Abstract

This study evaluated whether selected probiotic strains administered in-ovo could achieve performance and physiological outcomes comparable to those obtained with in-feed antibiotic growth promoters in broiler chickens. A preliminary in-ovo screening trial was conducted to identify probiotic strains and doses that did not negatively affect hatch performance. Based on this trial, two probiotic combinations were selected for an animal trial: a two-strain mixture (**Prob1**: *Bacillus subtilis* and *Enterococcus faecium*) and a four-strain mixture (**Prob2**: *E. faecium, Pediococcus acidilactici, Bifidobacterium animalis*, and *Lactobacillus reuteri*), both administered in-ovo on embryonic day 18 at 1.5 × 10⁷ CFU/egg. After hatch, birds were placed in a completely randomized block design and assigned to four treatments (*n* = 18 pens/group, 9 pens/sex, 45 birds/pen): negative control (**CTRL-**), antibiotic control (**CTRL+**, 0.5 kg bacitracin methylene disalicylate/ton of feed), and the two in-ovo probiotic treatments fed antibiotic-free diets. Growth performance, breast meat yield, meat quality traits, liver lipid peroxidation, and cecal short-chain fatty acids (**SCFA**) were evaluated. The mixed-effects model revealed that under the experimental conditions of this study, neither in-feed antibiotics nor in-ovo probiotics significantly affected final body weight, feed intake, or feed efficiency relative to CTRL- birds. However, Prob1 increased (*P* = 0.014) breast meat yield compared with CTRL+ birds. Prob1 exhibited higher (*P* < 0.001) breast meat ultimate pH and lower (*P* = 0.013) shear force than CTRL+. Both probiotic treatments reduced (*P* < 0.001) liver malondialdehyde concentrations compared with both control groups, indicating improved oxidative status. The Prob2 mixture increased (*P* < 0.038) cecal butyric acid concentration on d 10, although this effect did not persist at market age. Overall, Prob2 exhibited the most consistent effects on physiological and microbial-related parameters without affecting growth performance. These results indicate that neither in-ovo probiotics nor in-feed BMD act as classical growth promoters under controlled conditions but in-ovo probiotics may support bird health and resilience. Further validation under commercial production conditions is warranted.

## Introduction

Historically, sub-therapeutic doses of antibiotics, commonly referred to as antimicrobial growth promoters (**AGP**), were widely used in poultry production to improve feed efficiency and growth performance (Fernández Miyakawa et al., 2024; Graham et al., 2007). Although AGP were initially believed to act primarily by reducing intestinal pathogen load, recent research suggests that their growth-promoting effects are largely mediated through host-centered mechanisms, including modulation of intestinal inflammation, improvement of metabolic efficiency, and enhancement of intestinal barrier function (Fernández Miyakawa et al., 2024; Graham et al., 2007; Rafiq et al., 2022).

However, the extensive use of antibiotics in animal production has contributed to the emergence of antimicrobial resistance (**AMR**), which represents a major global public health concern (Church and McKillip, 2021; Djordjevic and Morgan, 2019; Rahman et al., 2022). As a result, many countries have implemented regulations restricting the preventive use of medically important antibiotics in poultry production, increasing the need for alternative strategies to maintain animal health and performance (Bean-Hodgins and Kiarie, 2021; Dimuccio et al., 2026).

In this context, probiotics have emerged as one of the most promising alternatives to AGP. These microorganisms can improve gut health through competitive exclusion of pathogens (Opazo et al., 2025; Sachdeva et al., 2025), as well as through modulation of intestinal microbiota composition and metabolic activity, leading to enhanced nutrient utilization and barrier function (Madej et al., 2025; Zhang et al., 2022). In addition, probiotics can exert immunomodulatory effects by regulating cytokine production and reducing intestinal inflammation, which may contribute to improved host resilience and performance (Obianwuna et al., 2023; Qiu et al., 2021). Emerging evidence also highlights links between gut microbiota, oxidative status, and peripheral tissues, including muscle metabolism and meat quality, suggesting that probiotic-mediated effects extend beyond the gastrointestinal tract to influence systemic physiological processes (Chen et al., 2023; He et al., 2025).

Numerous studies and meta-analyses have reported overall beneficial effects of probiotics on broiler performance, health, and meat quality. However, a substantial degree of variability in probiotic responses remains a consistent finding across the literature, which is often attributed to differences in probiotic strains, experimental conditions, and flock health status (Blajman et al., 2014; Opazo et al., 2025; Singh et al., 2025; Yaqoob et al., 2022; Yibar and Uzabaci, 2024). One important source of variability is that post-hatch probiotic administration attempts to modify an already developing and increasingly stable microbiota.

In this context, in-ovo administration of probiotics emerges as a biologically distinct alternative to post-hatch delivery. In-ovo delivery introduces probiotics into the amniotic fluid during late embryogenesis (typically embryonic days 17-19) and offers several advantages. First, beneficial bacteria can colonize the gastrointestinal tract prior to hatch, providing a competitive advantage against pathogens before chicks are exposed to the external environment (Roto et al., 2016). Second, unlike post-hatch administration, which attempts to modulate an already developing microbiota, in-ovo delivery supports gastrointestinal tract maturation in preparation for exogenous feeding, which enhances early nutrient absorption and feed efficiency immediately after placement (Gao et al., 2024; Leão et al., 2021; Madej et al., 2025; Roto et al., 2016). Third, in-ovo administration allows modulation of physiological processes that are limited or cease after hatch, such as hyperplastic muscle growth, through the upregulation of myogenic regulatory factors, resulting in increased muscle fiber density and relative breast muscle weight after hatch (Muyyarikkandy et al., 2023). Finally, in-ovo delivery relies on automated technologies that enable precise dosing across uniform embryo populations (Yehia et al., 2024), which reduces variability associated with inconsistent intake during post-hatch administration via feed or water and ensuring uniform exposure across individuals.

Therefore, the objective of the present study was to evaluate whether probiotics administered in-ovo could serve as an alternative to AGP in broiler production, using bacitracin methylene disalicylate (**BMD**) as a reference. Few studies have directly compared in-ovo probiotic administration with conventional in-feed antibiotic supplementation across multiple physiological and production parameters within the same experiment. In the present study, a preliminary screening phase was conducted to identify probiotic strains and doses compatible with embryonic development, after which selected probiotic combinations were evaluated for their effects on growth performance, meat yield, meat quality traits, oxidative stress indicators, and cecal fermentation profiles. This approach provides a comprehensive assessment of early microbial intervention as a strategy to support broiler performance and physiology in antibiotic-reduced production systems.

## Materials and methods

### Ethics statement

This study was conducted at the Deschambault Research Center in Animal Science (Deschambault, Quebec, Canada) in accordance with the guidelines of the Canadian Council on Animal Care and was approved by the Institutional Animal Care and Use Committee of Université Laval (protocol number 2024-1552).

### Initial screening of probiotic strains

The following probiotic species were selected for evaluation based on the literature and availability: *Bacillus subtilis, Lactobacillus helveticus, Lactobacillus reuteri, Bifidobacterium bifidum, Bifidobacterium animalis, Pediococcus acidilactici*, and *Enterococcus faecium*. All probiotics used in this study were obtained from Creative Biogenes (Shirley, NY, USA). Due to practical constraints on testing all possible strain combinations, seven formulations of live probiotics were prepared: (1) *B. subtilis*, (2) *L. helveticus*, (3) *B. bifidum*, (4) *E. faecium*, (5) a 1:1 mixture of *L. helveticus* and *B. bifidum*, (6) a 1:1 mixture of *B. subtilis* and *E. faecium*, and (7) a 1:1:1:1 mixture of *E. faecium, P. acidilactici, B. animalis*, and *L. reuteri*. These formulations were prepared in the sterile diluent (Boehringer Ingelheim Animal Health, Ontario, Canada) used at the hatchery to prepare in-ovo vaccination solutions. These species represent functionally diverse bacterial groups commonly used in probiotic formulations and are considered suitable candidates for early-life microbial intervention. Practical considerations, including strain availability and compatibility with in-ovo delivery conditions, were also taken into account.

Each formulation was administered in-ovo into the amniotic fluid of developing embryos on embryonic day 18 (ED18) at the following doses (CFU/egg): 0 (control, sterile diluent), 1 × 10^6^, 4 × 10^6^, 7 × 10^6^, 1 × 10^7^, 1.3 × 10^7^, and 1.5 × 10^7^. Probiotic suspensions were prepared 8 h prior to injection and maintained at 4 °C until injection to preserve bacterial viability.

For each formulation-dose combination, six hatching baskets were used, with 168 eggs per basket, resulting in 1,008 eggs per formulation-dose combination (*n* = 7 solutions × 7 doses × 6 replicates per combination × 168 eggs per replicate = 49,392 injected eggs in total). Fertile eggs were obtained from a single commercial breeder flock (Couvoir Scott, Scott, QC, Canada) and randomly assigned to treatment groups to minimize source-related variability. In-ovo injections were performed in combination with the Marek disease vaccine (PREVEXXION, Boehringer Ingelheim, Canada) using an automated injection system (Embrex Inovoject JW84 System, Zoetis, Canada) with a total injected volume of 52 µL/egg.

At hatch, the per basket number of chicks was recorded to calculate hatchability (*n* = 6 baskets/formulation-dose combination). In addition, five chicks per hatching basket were randomly selected (*n* = 30 chicks per formulation-dose combination) to assess chick quality. Individual body weight at hatch (**BW0**) and chick length, measured from the tip of the beak to the tip of the toe, were recorded.

Our approach allowed the assessment of potential additive or synergistic effects of combining bacterial species with different modes of action. Given the limited number of studies evaluating multi-strain probiotic administration in-ovo, particularly across a range of doses, this screening was designed to identify formulations that maintain hatch performance while providing a basis for subsequent evaluation under post-hatch conditions.

### Post-hatch experimental design and housing

Based on the hatch performance screening results, two formulation-dose combinations were selected for subsequent evaluation. The first consisted of a 1:1 mixture of *B. subtilis* and *E. faecium* administered at a dose of 1.5 × 10^7^ CFU/egg, and the second consisted of a 1:1:1:1 mixture of *E. faecium, P. acidilactici, B. animalis*, and *L. reuteri* administered at the same dose.

In total, three in-ovo treatment groups were established: a control group (**CTRL**, *n* = 1,620 eggs) injected with sterile diluent without probiotics, the two-strain probiotic group (**Pb1**, *n* = 810 eggs), and the four-strain probiotic group (**Pb2**, *n* = 810 eggs). All groups were also injected with the Marek disease vaccine.

On the day of hatch, chicks were counted, wing-sexed, vaccinated against infectious bronchitis by spray (Massachusetts-type strain), and placed in pre-identified transportation boxes according to treatment group replicates. Chicks were then transported to the Deschambault Research Center in Animal Science (CRSAD, Deschambault, QC, Canada) and allocated to a completely randomized block design with four treatments × two sexes factorial. Birds were assigned to one of four post-hatch treatments: (1) a negative control group (**CTRL-**, *n* = 810 chicks, *n* = 18 pens, 9 pens per sex, 45 chicks per pen) originating from control eggs and fed antibiotic-free standard corn-soybean meal starter (d 1 to d 10), grower (d 11 to d 21), and finisher (d 22 to d 31) broiler diets; (2) a positive control group (**CTRL+**, *n* = 810 chicks, *n* = 18 pens, 9 pens per sex, 45 chicks per pen) originating from control eggs and fed the same diets supplemented with BMD (0.5 kg/ton); (3) the two-strain probiotic group (**Prob1**, *n* = 810 chicks, *n* = 18 pens, 9 pens per sex, 45 chicks per pen), originating from Pb1 in-ovo treated eggs and fed antibiotic-free diets; and (4) the four-strain probiotic group (**Prob2**, *n* = 810 chicks, *n* = 18 pens, 9 pens per sex, 45 chicks per pen), originating from Pb2 in-ovo treated eggs and fed antibiotic-free diets. All diets contained Narasin–Nicarbazin (0.5 kg/ton) to control coccidiosis. Diet formulation and calculated nutrient content are given in supplementary Tables S1, S2, and S3.

Each treatment consisted of 18 pens (9 pens per sex), with 45 chicks per pen (approximately 31 kg/m^2^). Pens were bedded with fresh sawdust and equipped with nipple drinkers and manual circular feeders. Birds had *ad libitum* access to feed and water throughout the experiment.

The lighting program consisted of 23 h of light per day for the first 4 days post-hatch to allow chicks to locate feeders and drinkers, after which the photoperiod was gradually reduced to 18 h of light and 6 h of darkness (18L:6D) for the remainder of the experiment. Ambient temperature was maintained at 33 °C during the first week post-hatch, gradually reduced to 22 °C by the end of the third week, and maintained at 22 °C until the end of the experiment.

### Performance traits

Throughout the experimental period, pen-level body weight was recorded at placement (**BW1**) and on days 10 (**BW10**), 21 (**BW21**), and 31 (**BW31**). Feed intake was recorded per pen on days 10 (**FI10**), 21 (**FI21**), and 31 (**FI31**). Body weight gain (**BWG**), average daily gain (**ADG**), and feed conversion ratio (**FCR**) were calculated for the corresponding periods. For all these traits, the pen was considered the statistical unit.

### Meat quality traits and lipid peroxidation

On day 35, two birds per pen were randomly selected (*n* = 36 birds per treatment), individually weighed, and processed as described by Achour et al. (2025). The entire right *Pectoralis major* muscle was excised and weighed, and subsequently used to assess breast meat yield and technological quality traits (including the ultimate pH, color parameters, texture, and cooking loss with and without marination), following the procedures detailed in Achour et al. (2025).

In addition, tissue samples (*n* = 18 per treatment and tissue) were collected from the cranial portion of the left *Pectoralis major* muscle and from the liver to quantify lipid peroxidation by measuring the thiobarbituric acid-reactive substances (**TBARS**) index, following the protocol described by Askri et al. (2025). For these traits, the bird was considered the experimental unit.

### Short-chain fatty acid quantification

Short-chain fatty acid (SCFA) concentrations in cecal contents were determined by gas chromatography following acid extraction and purification procedures. Briefly, approximately 1 g of cecal content was sampled on d 10 and d 35 (*n* = 12 birds per group), weighed into a 15-mL tube, mixed with 2 mL of 1.5% sulfuric acid, vortexed for 1 min to ensure complete homogenization, resulting in a final dilution of 1:3, and stored at −20 °C until analysis. Prior to analysis, samples were centrifuged at 10,000 rpm for 15 min at 4°C, and the supernatant was collected. For purification, 1 mL of supernatant was transferred to a glass tube and 100 µL of 30 mM 2-ethylbutyric acid was added as an internal standard. Samples were mixed with AG 50WX8 resin (100–200 mesh, H form) to remove interfering ions, vortexed, and allowed to stand for 10 min. The supernatant was then filtered through a 0.2 μm polyethersulfone filter directly into gas chromatography vials fitted with inserts and sealed with PTFE septa. SCFA concentrations were determined using a gas chromatograph (Agilent 7820A, Agilent Technologies Canada Inc., Mississauga, ON, Canada) equipped with a flame ionization detector and a polar capillary column (HP-Innowax, 30 m length, 0.320 mm internal diameter, 0.25 µm film thickness). The injector split ratio was set to 25:1. The oven temperature was initially set at 80 °C and maintained for 0.5 min, then increased to 180 °C at 10 °C/min, followed by a second increase to 220 °C at 30 °C/min and held at 220 °C for 2 min. SCFA were expressed as a percentage of sample total SCFA. For SCFA, the bird was considered the experimental unit.

### Statistical analysis

Hatch performance data from the in-ovo screening experiment were analyzed using linear mixed-effects models fitted with the *lmerTest* package (version 3.2.1) in R (Kuznetsova et al., 2017). Formulation, dose, and their interaction were included as fixed effects, and hatching basket was included as a random effect. Marginal means were estimated using the *emmeans* package (version 2.2.2), and dose effects within each formulation were compared with the control using Dunnett's procedure.

Post-hatch data were analyzed using linear mixed-effects models with treatment, sex, and their interaction as fixed effects. Block was included as a random effect for pen-level data, and pen nested within block for bird-level data. Marginal means were compared using Tukey-adjusted *P*-values. Pre-planned contrasts were used to compare probiotic treatments relative to both the negative and antibiotic controls. Model assumptions were assessed by visual inspection of residual plots. Statistical significance was declared at *P* < 0.05, and tendencies were defined as 0.05 < *P* < 0.10.

## Results

### Effect of treatment on hatch performance

The effect of in-ovo treatments on hatchability is presented in Table 1. No significant differences were detected between treatment groups and their respective controls across the tested doses. However, the Prob2 group consistently exhibited numerically higher hatchability values relative to its control.

**Table 1:** Effects^1^ of in-ovo probiotic treatments and doses on hatchability and chick quality traits2.

Treatment^1^	Dose	Hatch traits			P-value^3^		
		Hatch (%)	BW0 (g)	Length (cm)	Hatch	BW	Length
B. subtilis	CTRL	91.5 (1.0)	40.5 (0.6)	19.2 (0.1)			
B. subtilis	1 × 10^6^	92.0 (1.0)	41.4 (0.6)	19.3 (0.1)	0.988	0.722	0.651
B. subtilis	4 × 10^6^	92.0 (1.0)	42.6 (0.6)	19.4 (0.1)	0.988	0.061	0.331
B. subtilis	7 × 10^6^	91.0 (1.0)	41.8 (0.6)	19.1 (0.1)	0.988	0.367	0.956
B. subtilis	10 × 10^6^	90.6 (1.0)	42.4 (0.6)	19.1 (0.1)	0.937	0.071	0.986
B. subtilis	1.3 × 10^7^	92.6 (1.0)	42.7 (0.6)	19.3 (0.1)	0.892	0.036	0.840
B. subtilis	1.5 × 10^7^	90.7 (1.0)	42.8 (0.6)	19.3 (0.1)	0.955	0.026	0.852
L. helveticus	CTRL	92.5 (1.0)	41.9 (0.6)	18.7 (0.1)			
L. helveticus	1 × 10^6^	90.1 (1.0)	42.9 (0.6)	18.9 (0.1)	0.377	0.708	0.582
L. helveticus	4 × 10^6^	90.7 (1.0)	42.9 (0.6)	18.5 (0.1)	0.635	0.660	0.390
L. helveticus	7 × 10^6^	94.1 (1.0)	39.9 (0.6)	18.8 (0.1)	0.678	0.070	0.895
L. helveticus	10 × 10^6^	92.3 (1.0)	41.3 (0.6)	18.9 (0.1)	0.999	0.906	0.815
L. helveticus	1.3 × 10^7^	92.8 (1.0)	42.8 (0.6)	18.7 (0.1)	0.997	0.754	0.952
L. helveticus	1.5 × 10^7^	92.9 (1.0)	42.2 (0.6)	18.6 (0.1)	0.994	0.994	0.885
B. bifidum	CTRL	92.8 (1.0)	41.3 (0.6)	18.8 (0.1)			
B. bifidum	1 × 10^6^	90.2 (1.0)	43.4 (0.6)	18.5 (0.1)	0.302	0.071	0.039
B. bifidum	4 × 10^6^	91.6 (1.0)	42.6 (0.6)	18.3 (0.1)	0.863	0.442	0.000
B. bifidum	7 × 10^6^	90.7 (1.0)	42.1 (0.6)	18.4 (0.1)	0.503	0.827	0.010
B. bifidum	10 × 10^6^	91.7 (1.0)	41.2 (0.6)	18.9 (0.1)	0.892	0.998	0.999
B. bifidum	1.3 × 10^7^	92.0 (1.0)	41.5 (0.6)	18.8 (0.1)	0.955	0.997	0.976
B. bifidum	1.5 × 10^7^	92.2 (1.0)	43.7 (0.6)	18.5 (0.1)	0.980	0.031	0.060
E. faecium	CTRL	92.8 (1.0)	41.9 (0.6)	18.8 (0.1)			
E. faecium	1 × 10^6^	92.8 (1.0)	43.1 (0.6)	18.6 (0.1)	0.999	0.517	0.265
E. faecium	4 × 10^6^	92.2 (1.0)	42.3 (0.6)	18.8 (0.1)	0.980	0.976	0.913
E. faecium	7 × 10^6^	92.7 (1.0)	40.8 (0.6)	18.9 (0.1)	0.999	0.535	0.999
E. faecium	10 × 10^6^	91.2 (1.0)	42.0 (0.6)	18.3 (0.1)	0.720	0.999	0.000
E. faecium	1.3 × 10^7^	92.7 (1.0)	42.7 (0.6)	18.3 (0.1)	0.999	0.761	0.000
E. faecium	1.5 × 10^7^	91.8 (1.0)	41.9 (0.7)	18.5 (0.1)	0.916	0.999	0.042
LH + BB	CTRL	91.9 (1.0)	41.3 (0.6)	19.2 (0.1)			
LH + BB	1 × 10^6^	93.1 (1.0)	42.1 (0.6)	19.2 (0.1)	0.863	0.851	0.986
LH + BB	4 × 10^6^	92.2 (1.0)	42.3 (0.6)	18.9 (0.1)	0.999	0.732	0.135
LH + BB	7 × 10^6^	90.9 (1.0)	41.5 (0.6)	19.1 (0.1)	0.916	0.999	0.944
LH + BB	10 × 10^6^	91.6 (1.0)	42.8 (0.6)	19.1 (0.1)	0.999	0.347	0.800
LH + BB	1.3 × 10^7^	91.1 (1.0)	42.4 (0.6)	19.1 (0.1)	0.955	0.659	0.851
LH + BB	1.5 × 10^7^	92.3 (1.0)	41.9 (0.6)	19.1 (0.1)	0.994	0.929	0.967
BS + EF	CTRL	91.9 (1.0)	42.1 (0.6)	19.2 (0.1)			
BS + EF	1 × 10^6^	93.1 (1.0)	41.0 (0.6)	19.0 (0.1)	0.863	0.630	0.714
BS + EF	4 × 10^6^	94.5 (1.0)	42.5 (0.6)	19.0 (0.1)	0.268	0.946	0.437
BS + EF	7 × 10^6^	90.3 (1.0)	43.1 (0.6)	19.1 (0.1)	0.720	0.659	0.999
BS + EF	10 × 10^6^	93.5 (1.0)	41.7 (0.6)	19.0 (0.1)	0.720	0.969	0.585
BS + EF	1.3 × 10^7^	91.9 (1.0)	41.6 (0.6)	19.1 (0.1)	0.999	0.958	0.828
BS + EF	1.5 × 10^7^	91.6 (1.0)	41.1 (0.6)	19.1 (0.1)	0.997	0.691	0.885
EF + PA + BA + LR	CTRL	91.5 (1.0)	42.2 (0.6)	19.2 (0.1)			
EF + PA + BA + LR	1 × 10^6^	91.9 (1.0)	42.0 (0.6)	19.1 (0.1)	0.994	0.999	0.990
EF + PA + BA + LR	4 × 10^6^	91.9 (1.0)	42.4 (0.6)	19.3 (0.1)	0.994	0.991	0.802
EF + PA + BA + LR	7 × 10^6^	91.3 (1.0)	43.8 (0.6)	19.2 (0.1)	0.992	0.244	0.999
EF + PA + BA + LR	10 × 10^6^	92.7 (1.0)	42.6 (0.6)	19.1 (0.1)	0.863	0.968	0.873
EF + PA + BA + LR	1.3 × 10^7^	90.6 (1.0)	42.2 (0.6)	19.2 (0.1)	0.937	0.999	0.980
EF + PA + BA + LR	1.5 × 10^7^	92.0 (1.0)	44.2 (0.6)	19.3 (0.1)	0.988	0.079	0.863

1 B. subtilis: Bacillus subtilis, L. helveticus: Lactobacillus helveticus, B. bifidum: Bifidobacterium bifidum, LH: L. helveticus, BB: B. bifidum, BBS:B. subtilis, EF: E. faecium, PAPA:ediococcus acidilactici, BA: Bifidobacterium animalis, LR: Lactobacillus reuteri.

2 Hatch: hatchability, BW0: Chick body weight at hatch, length: Chick body length between the tip of the beak and the middle toe.

3 Values are estimated marginal means and their standard errors (SE). Pairwise comparisons against CTRL were performed using Dunnett’s adjustment.

For BW0, treatment effects were limited. Single-strain treatments such as *B. subtilis* and *B. bifidum* resulted in significantly higher BW0 compared with their respective controls, while the Prob2 group showed a tendency (*P* < 0.10) toward increased BW0 at the highest dose (Table 1).

Body length at hatch was significantly affected by treatment (Table 1). Reductions in body length were observed in *B. bifidum* at intermediate doses and *E. faecium* at higher doses. In contrast, the Prob2 treatment exhibited body length values comparable to or numerically greater than the control across doses, indicating no adverse effect on this trait.

Overall, the Prob2 group exhibited the most consistent numerical advantages across hatchability and chick quality traits without evidence of adverse effects, whereas other formulations showed more variable responses.

### Effect of treatment on post-hatch growth performance

The results of the statistical analysis are presented in Table 2. At the end of the starter phase, contrast analysis indicated that birds in the Prob2 group had lower BW than those in CTRL- (C3, *P* = 0.043) and CTRL+ (C5, *P* = 0.043). In contrast, BW of Prob1 birds did not differ from either CTRL- (C2, *P* = 0.538) or CTRL+ (C4, *P* = 0.538). Body weight was also similar between CTRL- and CTRL+ (C1, *P* = 0.935). During the grower phase, treatment effects on BW remained significant. Specifically, both in-ovo probiotic groups exhibited lower BW compared with CTRL- (C2 and C3, *P* = 0.024 and *P* = 0.025 for Prob1 and Prob2, respectively) and CTRL+ (C4 and C5, *P* < 0.001 for Prob1 and Prob2, respectively), whereas CTRL- and CTRL+ did not differ (C1, *P* = 0.115). By d 31, treatment no longer affected BW (*P* = 0.142). A similar pattern was observed for ADG (Table 2), and ADG measured over the entire experimental period (d 0 to d 31) was not influenced by treatment (*P* = 0.134).

**Table 2:** Effect of treatment^1^, sex, and their interaction on growth performance2.

											Treatment											
	BW (g)			ADG (g/b/d)							FI (g/b)					FCR						
Age (d)	10	21	31	10	21		31		0 - 31		10	21		31	0 – 31	10	21			31	0 - 31	
CTRL-	318	1108	2253	27.3		71.5		113		71.2	276		984	1605	2865	1.014	1.257			1.418	1.308	
CTRL+	317	1122	2277	27.4		72.8		113		72.0	277		988	1547	2812	1.011	1.235			1.373	1.276	
Prob1	312	1084	2230	26.7		69.3		111		70.4	265		957	1546	2769	0.994	1.256			1.388	1.29	
Prob2	305	1083	2238	26.2		70.3		113		70.7	267		968	1594	2829	1.022	1.253			1.405	1.303	
Pooled SE	3.63	10.17	17.98	0.380		0.780		1.44		0.580	3.16		10.3	26.5	28.0	0.014	0.010			0.010	0.009	
											Sex											
Males	323	1171	2429	27.9		76.5		122		76.9	278		1028	1674	2980	1.02	1.27		1.36			1.26
Females	303	1028	2070	25.8		65.4		103		65.3	264		920	1472	2658	0.997	1.22		1.43		1.32	
Pooled SE	2.89	9.21	14.8	0.031		0.680		1.070		0.470	2.46		7.87	20.32	21.41	0.010	0.008		0.010		0.006	
											P-value											
TRT	0.027	< 0.001	0.142	0.039		< 0.001		0.723		0.134	0.009		0.098	0.213	0.087	0.384		0.465	0.222			0.095
Sex	< 0.001	< 0.001	< 0.001	< 0.001		< 0.001		< 0.001		< 0.001	< 0.001		< 0.001	< 0.001	< 0.001	0.038	< 0.001		< 0.001			< 0.001
I	0.082	0.062	0.457	0.078		0.317		0.695		0.463	0.490		0.461	0.210	0.306	0.280	0.413		0.021			0.121
											Contrasts^3^											
C1	0.935	0.115	NS	0.87		0.22		NA		NS	0.916		NS	NS	NS	NS	NS			NS	NS	
C2	0.528	0.024	NS	0.52		0.02		NA		NS	0.040		NS	NS	NS	NS	NS			NS	NS	
C3	0.043	0.025	NS	0.07		0.22		NA		NS	0.068		NS	NS	NS	NS	NS			NS	NS	
C4	0.538	0.000	NS	0.52		0.00		NA		NS	0.038		NS	NS	NS	NS	NS			NS	NS	
C5	0.043	0.000	NS	0.06		0.01		NA		NS	0.068		NS	NS	NS	NS	NS			NS	NS	

1 CTRL-: birds fed the negative control diet, CTRL+: birds fed bacitracin (0.5 kg/t)-supplemented diet, Prob1: birds hatched from eggs injected with a mixture of two probiotic strains and fed the CTRL- diet, Prob2: birds hatched from eggs injected with a mixture of four probiotic strains and fed the CTRL- diet.

2 BW: Body weight, ADG: Average daily gain, FI: Feed intake, FCR: Feed conversion ratio.

3 Treatment contrasts: C1: effect of bacitracin (CTRL+ vs CTRL-), C2: effect of the two probiotic strains mixture (Prob1 vs CTRL-), C3: effect of the four probiotic strains mixture (Prob2 vs CTRL-), C4: effect of the two probiotic strains mixture relative to bacitracin (Prob1 vs CTRL+), C5: effect of the four probiotic strains mixture relative to bacitracin (Prob2 vs CTRL+).

Feed intake was significantly affected by treatment at the end of the starter phase (Table 2). Birds in the Prob1 group consumed less feed than those in CTRL- (C2, *P* = 0.040), whereas Prob2 birds tended to show lower FI than CTRL- (C3, *P* = 0.068). A similar pattern was observed relative to CTRL+, with reduced FI in Prob1 (C4, *P* = 0.038) and a tendency toward lower FI in Prob2 (C5, *P* = 0.068). Feed intake did not differ between CTRL- and CTRL+ (C1, *P* = 0.916). No treatment effects were detected during the grower or finisher phases (*P* = 0.098 and *P* = 0.213, respectively), although FI over the entire experimental period tended to differ among treatments with birds in the Prob1 group numerically having the lowest FI (*P* = 0.087). Treatment did not affect FCR at any growth phase (Table 2), although a tendency toward improved overall FCR in CTRL+ birds was observed (*P* = 0.095).

Regarding sex effects, males exhibited greater BW, ADG, and FI, as well as lower FCR, than females throughout the experimental period (Table 2).

### Effect of treatment on breast meat yield

Body weight at slaughter did not differ among treatment groups (Table 3). However, BMY was influenced by the treatment, with Prob1 birds showing higher BMY compared with CTRL+ (C4, *P* = 0.014). Similarly, the yield of the *Pectoralis major* muscle was greater in Prob1 than in CTRL+ (C4, *P* = 0.025), whereas all other contrasts were not significant. On the contrary, the yield of the *Pectoralis minor* muscle was not affected by treatment.

**Table 3:** Effect of treatment^1^, sex, and their interaction on body weight at slaughter and breast meat yield2.

	BW (g)	Breast yield (%)		
		BMY	P. Major	P. Minor
	Treatment			
CTRL-	2690	22.3	19.3	3.02
CTRL+	2701	21.6	18.5	3.06
Prob1	2652	22.9	19.7	3.22
Prob2	2672	22.0	18.9	3.12
Pooled SE	31.2	0.310	0.290	0.080
	Sex			
Males	2873	21.7	18.8	2.90
Females	2484	22.8	19.4	3.30
Pooled SE	21.9	0.210	0.200	0.060
	P-value			
TRT	0.696	0.024	0.030	0.390
Sex	< 0.001	< 0.001	0.023	< 0.001
I	0.203	0.095	0.103	0.874
	Contrasts^3^			
C1	NS	0.498	0.343	NS
C2	NS	0.498	0.743	NS
C3	NS	0.657	0.743	NS
C4	NS	0.014	0.025	NS
C5	NS	0.657	0.743	NS

1 CTRL-: birds fed the negative control diet, CTRL+: birds fed bacitracin (0.5 kg/t)-supplemented diet, Prob1: birds hatched from eggs injected with a mixture of two probiotic strains and fed the CTRL- diet, Prob2: birds hatched from eggs injected with a mixture of four probiotic strains and fed the CTRL- diet.

2 BW: Body weight, BMY: Breast meat yield, P. Major: Yield of the *Pectoralis major* muscle, P. Minor: Yield of the *Pectoralis minor* muscle.

3 Treatment contrasts: C1: effect of bacitracin (CTRL+ vs CTRL-), C2: effect of the two probiotic strains mixture (Ptob1 vs CTRL-), C3: effect of the four probiotic strains mixture (Prob2 vs CTRL-), C4: effect of the two probiotic strains mixture relative to bacitracin (Prob1 vs CTRL+), C5: effect of the four probiotic strains mixture relative to bacitracin (Prob2 vs CTRL+).

Regarding sex effects, males exhibited greater BW at slaughter (*P* < 0.001), but lower BMY (*P* < 0.001) and lower *P. major* yield (*P* = 0.023) compared with females. The yield of the *P. minor* muscle was also lower in males than in females (*P* < 0.001).

### Effect of treatment on breast meat quality

The treatment effect on breast muscle pHu was significant (Table 4). Birds in the Prob1 group exhibited higher pHu values than those in CTRL- (C2, *P* = 0.035) and CTRL+ (C4, *P* < 0.001). In contrast, Prob2 birds only tended to have higher pHu than CTRL+ (C5, *P* = 0.066) and did not differ from CTRL- (C3, *P* = 0.412). No differences were observed between CTRL- and CTRL+ (C1, *P* = 0.412).

**Table 4:** Effect of treatment^1^, sex, and their interaction on breast meat ultimate pH and color parameters2.

	pHu	L*	a*	b*
	Treatment			
CTRL-	5.82	61.6	6.28	14.8
CTRL+	5.79	62.4	5.89	14.7
Prob1	5.89	62.5	6.52	15.5
Prob2	5.85	63.8	6.24	16.8
Pooled SE	0.016	0.502	0.537	0.419
	Sex			
Males	5.88	61.7	6.14	14.5
Females	5.80	63.5	6.33	16.1
Pooled SE	0.011	0.356	0.375	0.292
	P-value			
TRT	0.001	0.028	0.871	< 0.001
Sex	< 0.001	< 0.001	0.724	< 0.001
I	0.770	0.034	0.296	0.528
	Contrasts^3^			
C1	0.412	0.597	NS	0.422
C2	0.035	0.587	NS	0.080
C3	0.412	0.015	NS	0.000
C4	0.000	0.885	NS	0.297
C5	0.066	0.244	NS	0.000

1 CTRL-: birds fed the negative control diet, CTRL+: birds fed bacitracin (0.5 kg/t)-supplemented diet, Prob1: birds hatched from eggs injected with a mixture of two probiotic strains and fed the CTRL- diet, Prob2: birds hatched from eggs injected with a mixture of four probiotic strains and fed the CTRL- diet.

2 pHu: The ultimate pH of the *Pectoralis major* muscle, L*: lightness index, a*: redness index, b*: yellowness index of the *Pectoralis major* muscle.

3 Treatment contrasts: C1: effect of bacitracin (CTRL+ vs CTRL-), C2: effect of the two probiotic strains mixture (Ptob1 vs CTRL-), C3: effect of the four probiotic strains mixture (Prob2 vs CTRL-), C4: effect of the two probiotic strains mixture relative to bacitracin (Prob1 vs CTRL+), C5: effect of the four probiotic strains mixture relative to bacitracin (Prob2 vs CTRL+).

Treatment also influenced meat lightness (L*) (*P* = 0.028), with Prob2 birds exhibiting higher L* values than CTRL- (C3, *P* = 0.015), whereas all other contrasts were not significant. The redness parameter (a*) was not affected by treatment. On the contrary, yellowness (b*) was significantly affected (Table 4), with Prob2 birds showing higher b* values than both CTRL- (C3, *P* < 0.001) and CTRL+ (C5, *P* < 0.001). Birds in the Prob1 group tended to exhibit higher b* values than those in CTRL- (C2, *P* = 0.080), while CTRL- and CTRL+ did not differ (C1, *P* = 0.422).

Regarding sex effects, males exhibited higher breast muscle pHu and lower L* and b* values than females, whereas a* values were not affected by sex (Table 4).

The effects of treatment on freeze-thaw loss (FTL), cooking loss (CL), and Warner-Bratzler shear force (WBSF) are summarized in Table 5. Freeze-thaw loss was not affected by treatment (*P* = 0.304). In unmarinated breast meat, both CL and WBSF were significantly influenced by treatment. Cooking loss was slightly but significantly greater in Prob1 and Prob2 compared with CTRL- (C2 and C3, *P* = 0.005 and *P* = 0.022, respectively), whereas neither probiotic treatment differed from CTRL+ (C4 and C5, *P* = 0.172 and *P* = 0.324, respectively). CL also did not differ between CTRL- and CTRL+ (C1, *P* = 0.324). For WBSF, Prob1 and Prob2 exhibited lower values than CTRL+ (C4 and C5, *P* = 0.013 and *P* = 0.005, respectively), while neither differed from CTRL- (C2 and C3, *P* = 0.228). No difference was observed between CTRL- and CTRL+ (C1, *P* = 0.225).

**Table 5:** Effect of treatment^1^, sex, and their interaction on meat quality traits^2^ and lipid peroxidation.

	FTL (%)	Unmarinated		Marinated		TBARS (µg/g)	
		CL (%)	WBSF (N/cm2)	CL (%)	WBSF (N/cm2)	Liver	Breast
	Treatment						
CTRL-	11.7	23.8	24.1	20.6	18.9	0.825	0.320
CTRL+	9.81	24.6	28.9	22.9	22.4	1.12	0.314
Prob1	9.71	25.7	20.9	23.1	18.6	0.486	0.316
Prob2	9.85	25.4	19.9	23.7	18.4	0.473	0.323
Pooled SE	0.887	0.400	1.890	0.670	0.970	0.060	0.006
	Sex						
Males	11.0	25.0	23.0	23.0	19.7	0.736	0.316
Females	9.50	24.8	23.9	22.2	19.5	0.714	0.320
Pooled SE	0.633	0.293	1.35	0.479	0.684	0.049	0.005
	P-value						
TRT	0.304	0.000	0.000	0.006	0.012	0.000	0.825
Sex	0.080	0.580	0.636	0.205	0.780	0.718	0.565
I	0.868	0.819	0.253	0.317	0.140	0.378	0.324
	Contrasts^3^						
C1	NS	0.324	0.225	0.045	0.038	0.000	NS
C2	NS	0.005	0.228	0.035	0.999	0.000	NS
C3	NS	0.022	0.228	0.006	0.999	0.000	NS
C4	NS	0.172	0.013	0.846	0.023	0.000	NS
C5	NS	0.324	0.005	0.810	0.021	0.000	NS

1 CTRL-: birds fed the negative control diet, CTRL+: birds fed bacitracin (0.5 kg/t)-supplemented diet, Prob1: birds hatched from eggs injected with a mixture of two probiotic strains and fed the CTRL- diet, Prob2: birds hatched from eggs injected with a mixture of four probiotic strains and fed the CTRL- diet.

2 FTL: freeze – thaw loss, CL: cooking loss, WBSF: Warner-Batzler shear force, TBARS: Thiobarbituric acid reactive substances index.

3 Treatment contrasts: C1: effect of bacitracin (CTRL+ vs CTRL-), C2: effect of the two probiotic strains mixture (Ptob1 vs CTRL-), C3: effect of the four probiotic strains mixture (Prob2 vs CTRL-), C4: effect of the two probiotic strains mixture relative to bacitracin (Prob1 vs CTRL+), C5: effect of the four probiotic strains mixture relative to bacitracin (Prob2 vs CTRL+).

In marinated breast meat, both CL and WBSF were also affected by treatment (Table 5). CL was higher in Prob1 and Prob2 than in CTRL- (C2 and C3, *P* = 0.035 and *P* = 0.006, respectively), whereas values did not differ from CTRL+ (C4 and C5, *P* = 0.846 and *P* = 0.810, respectively). Warner-Bratzler shear force was lower in Prob1 and Prob2 than in CTRL+ (C4 and C5, *P* = 0.023 and *P* = 0.021, respectively) but did not differ from CTRL- (C2 and C3, *P* = 0.999).

Lipid peroxidation results are presented in Table 5. Treatment affected malondialdehyde concentrations in liver samples but not in breast muscle. Liver MDA concentrations were lower in both probiotic groups compared with CTRL- and CTRL+ (*P* < 0.001). In addition, CTRL+ birds showed higher liver MDA concentrations than CTRL- birds (*P* < 0.001).

Sex did not influence FTL, CL, or WBSF in either marinated or unmarinated breast meat (Table 5). Finally, males and females did not differ in terms of MDA concentrations in the liver (*P* = 0.718) or breast muscle (*P* = 0.565), as can be seen in Table 5.

### Effect of treatment on cecal short chain fatty acids and cecal pH

Cecal fermentation results are presented in Table 6. Treatment influenced cecal SCFA concentrations, particularly during the starter phase.

**Table 6:** Effect of treatment^1^, sex, and their interaction on cecal volatile fatty acids (VFA) and pH.

	Age (d)						
	10			35			
	VFA (%)			VFA (%)			pH
	Butyric	Propionic	Acetic	Butyric	Propionic	Acetic	
	Treatment						
CTRL-	11.9	1.82	85.0	15.3	4.4	77.8	5.91
CTRL+	10.5	2.08	85.7	14.7	5.3	77.1	6.10
Prob1	13.4	2.87	81.4	17.1	6.3	73.4	6.03
Prob2	14.3	2.57	81.3	18.8	4.9	73.5	5.74
Pooled SE	1.04	0.369	1.02	1.230	0.613	1.07	0.150
	Sex						
Males	12.3	2.46	83.6	16.5	5.0	75.6	6.01
Females	12.7	2.21	83.2	16.4	5.4	75.3	5.88
Pooled SE	0.705	0.245	0.692	0.890	0.439	0.779	0.110
	P-value						
TRT	0.029	0.135	0.001	0.090	0.179	0.005	0.30
Sex	0.656	0.464	0.652	0.901	0.449	0.787	0.34
I	0.217	0.845	0.078	0.184	0.539	0.226	0.85
	Contrasts^2^						
C1	0.542	NS	0.626	NS	NS	0.631	NS
C2	0.542	NS	0.039	NS	NS	0.029	NS
C3	0.281	NS	0.039	NS	NS	0.029	NS
C4	0.129	NS	0.012	NS	NS	0.057	NS
C5	0.038	NS	0.012	NS	NS	0.057	NS

1 CTRL-: birds fed the negative control diet, CTRL+: birds fed bacitracin (0.5 kg/t)-supplemented diet, Prob1: birds hatched from eggs injected with a mixture of two probiotic strains and fed the CTRL- diet, Prob2: birds hatched from eggs injected with a mixture of four probiotic strains and fed the CTRL- diet.

2 Treatment contrasts: C1: effect of bacitracin (CTRL+ vs CTRL-), C2: effect of the two probiotics strains mixture (Ptob1 vs CTRL-), C3: effect of the four probiotic strains mixture (Prob2 vs CTRL-), C4: effect of the two probiotic strains mixture relative to bacitracin (Prob1 vs CTRL+), C5: effect of the four probiotic strains mixture relative to bacitracin (Prob2 vs CTRL+).

At d 10, treatment significantly affected cecal butyric acid concentration. Birds in the Prob2 group exhibited higher butyric acid concentrations than those in CTRL+ (C5, *P* = 0.038), whereas values did not differ from CTRL- (C3, *P* = 0.281). Butyric acid concentrations in Prob1 birds did not differ from either CTRL- (C2, *P* = 0.542) or CTRL+ (C4, *P* = 0.129). No difference was observed between CTRL- and CTRL+ (C1, *P* = 0.542).

Treatment also influenced cecal acetic acid concentration at d 10. Both Prob1 and Prob2 birds exhibited lower acetic acid concentrations than CTRL- (C2 and C3, *P* = 0.039) and CTRL+ (C4 and C5, *P* = 0.012), whereas CTRL- and CTRL+ did not differ (C1, *P* = 0.626).

At d 35, treatment effects were observed only for cecal acetic acid concentration (Table 5). Prob1 and Prob2 birds had lower acetic acid concentrations than CTRL- (C2 and C3, *P* = 0.029) and tended to have lower values than CTRL+ (C4 and C5, *P* = 0.057). No difference was detected between CTRL- and CTRL+ (C1, *P* = 0.631). Cecal pH measured at d 35 was not affected by treatment.

Sex did not influence cecal short chain fatty acid concentrations or cecal pH in the present study.

## Discussion

The present study evaluated whether in-ovo administration of probiotic mixtures could achieve performance outcomes comparable to those obtained with in-feed antibiotic supplementation, with the broader objective of assessing their potential as an alternative strategy for antibiotic use in broiler production.

Any early nutritional intervention intended for in-ovo delivery must not adversely affect hatch performance traits of economic importance to hatcheries. Based on the screening results, treatments were selected using two criteria: (1) absence of negative effects on hatchability, BW0, and body length relative to the control, and (2) consistency of responses across traits and doses.

The four-strain formulation (Prob2), administered at the highest dose, was retained for further evaluation as it consistently maintained hatchability and chick quality traits at levels comparable to or greater than the control, without evidence of adverse effects. In addition, the two-strain combination of *Bacillus subtilis* and *Enterococcus faecium* (Prob1) was selected, as it showed consistently higher hatchability across doses while maintaining BW0 and body length, and represented the most stable response among the two-strain formulations evaluated.

Multi-strain mixtures were prioritized, as they may promote more effective microbiota modulation than single-strain approaches (Lambo et al., 2021). Previous studies have shown that in-ovo probiotic administration is generally safe, with minimal to no impact on hatchability and chick quality (Duan et al., 2021; Gao et al., 2024), which is consistent with our findings.

### Effect of treatment on growth performance

Growth performance results indicated that in-ovo probiotic administration in our study resulted in performance comparable to antibiotic supplementation, while neither treatment consistently differed from the negative control. This may be explained by the controlled experimental conditions, where the absence of environmental stress or disease pressure can limit the growth-promoting effects of feed additives (Siwek et al., 2018; Soumeh et al., 2021).

The transient differences in growth performance observed during the starter and grower phases, followed by the disappearance of treatment effects by d 31, suggest that early-life interventions primarily influence developmental processes rather than sustaining long-term improvements in growth. The lower BW observed during early growth in probiotic-treated birds may reflect increased metabolic demands associated with early microbial colonization, intestinal development, and immune system maturation (Castañeda et al., 2020; Dunislawska et al., 2017; Shehata et al., 2022; Wishna-Kadawarage et al., 2024; Chen et al., 2026). As birds mature and their microbiota stabilizes, compensatory growth may occur, resulting in similar growth performance among treatments at market age.

Overall, the absence of growth-promoting effects across all treatments suggests that our experimental conditions likely did not impose sufficient environmental or health challenges to reveal differences among interventions. Under such low-stress conditions, the baseline performance of birds may already be near optimal, limiting the potential for additional improvements. Future studies should evaluate the effect of these interventions under commercial conditions to validate their role in improving or maintaining performance.

### Effect of treatment on breast meat yield

Although treatment did not influence body weight at slaughter, differences were observed in breast meat yield. Birds receiving the Prob1 treatment exhibited greater BMY and *Pectoralis major* yield compared with antibiotic-fed birds, while no differences were detected relative to the negative control. Similar improvements in breast yield following in-ovo probiotic administration have been previously reported (Hossain et al., 2024; Tang et al., 2021)

The capacity of probiotics to match or exceed antibiotic-associated responses in breast muscle yield may be related to their influence on early muscle development. In-ovo probiotic administration has been shown to increase myofiber density and upregulate myogenic regulatory factors during embryonic development, suggesting enhanced muscle hyperplasia (Muyyarikkandy et al., 2023). In addition, probiotic administration may influence muscle energy metabolism during late embryogenesis, reducing muscle protein breakdown during hatching and favoring muscle accretion during post-hatch growth (Gao et al., 2025). These developmental adaptations may explain the increased breast meat yield observed in the present study despite similar final body weight among treatments.

### Effect of treatment on breast meat quality

In the present study, in-ovo probiotic administration resulted in measurable, yet trait-specific, effects on meat quality parameters, whereas antibiotic supplementation had no detectable influence on these traits. Notably, birds receiving the Prob1 treatment exhibited higher pHu, a parameter generally associated with improved water-holding capacity and reduced incidence of pale, soft, exudative (PSE)-like conditions (Alnahhas et al., 2014, 2015). However, this potential benefit was not consistently reflected across related traits, as both probiotic treatments were associated with increased cooking loss. This apparent discrepancy suggests that the effects of early-life probiotic interventions on muscle physiology may involve multiple, partially independent mechanisms regulating post-mortem water distribution and protein denaturation, which requires further research to confirm.

These findings are in line with the literature indicating that in-ovo administration of probiotics and synbiotics has a largely neutral or mildly modulating effect on the breast meat quality of broiler chickens (Ren et al., 2025; Tavaniello et al., 2023). While major deterioration of meat quality is avoided, specific functional changes in texture, chemical composition, and physico-chemical properties can occur depending on the specific bacterial strains and bioactives utilized (Tavaniello et al., 2023; Wishna-Kadawarage et al., 2024).

The lack of effect of antibiotic treatment on pHu, color parameters, and water-holding capacity is consistent with previous research indicating that antimicrobial growth promoters do not significantly alter core physicochemical meat quality traits compared with negative control diets (Soumeh et al., 2021).

In contrast, the observed reduction in shear force in probiotic-treated birds suggests a potential improvement in meat tenderness. This effect may be related to probiotic-induced changes in muscle structure, as probiotic supplementation has been associated with reduced myofiber diameter and cross-sectional area, traits that are negatively correlated with shear force (Xue et al., 2022), leading to a more tender meat.

Overall, in-ovo probiotics appear to have limited effects on most meat quality traits under normal rearing conditions but may influence specific muscle characteristics.

### Effect of treatment on oxidative status

Probiotic treatments reduced lipid peroxidation in liver tissue, indicating a systemic effect on oxidative status, although this effect was not observed in breast muscle. The liver is a major metabolic hub and plays a central role in lipid metabolism, supplying lipids to peripheral tissues and to the liver itself (Zaefarian et al., 2019). Therefore, this organ is likely subjected to higher oxidative pressure than breast muscle, even in the absence of a specific challenge, as was the case in the present study. Previous work using a mixture of *B. subtilis* in broilers exposed to chronic heat stress showed a significant reduction in lipid peroxidation in breast muscle (Cramer et al., 2018), while *Lactobacillus* spp. have been reported to reduce systemic oxidative stress and enhance antioxidant capacity in broilers exposed to mycotoxins (de Souza et al., 2020). These effects are often attributed to probiotic-induced increases in antioxidant enzyme activity, modulation of gut microbiota-derived metabolites, and reduced systemic inflammation (Herich et al., 2025). Together, these findings suggest that probiotics may modulate both muscle and systemic oxidative status, but that their effects under standard rearing conditions are less pronounced in the muscle than under stress or challenge conditions.

### Effect of treatment on cecal volatile fatty acids

Short-chain fatty acids are microbial metabolites produced through fermentation in the ceca and play important roles in gut health, immunity, and intestinal homeostasis (Ali et al., 2022; Liu et al., 2021).

In the present study, in-ovo probiotic administration influenced cecal fermentation profiles, particularly during early life. At d 10, birds receiving the four-strain Prob2 mixture exhibited higher cecal butyric acid and lower acetic acid concentrations compared with the antibiotic control, whereas by d 35 overall fermentation profiles were similar among treatments.

The lack of differences in cecal pH suggests that probiotic treatments did not increase total fermentation activity but rather altered microbial metabolic output, which is consistent with previous findings showing that AGP and their alternatives can modify microbiota function rather than composition (Hodak et al., 2023).

The strains included in the Prob2 mixture are known to promote SCFA production indirectly through microbial cross-feeding mechanisms. For example, *E. faecium* has been reported to promote SCFA-producing bacteria (Wang et al., 2020), while *P. acidilactici* produces lactic acid that can be utilized by other bacteria to produce SCFA (Jazi et al., 2018). The transient increase in butyric acid at d 10 relative to the antibiotic control could therefore be attributed to these SCFA-promoting effects. However, as the gut microbiota stabilizes with age, typically around d 21 (Zhou et al., 2021), the influence of both antibiotic and probiotic treatments on microbial fermentation profiles may diminish (Jia et al., 2022).

Interestingly, birds in the Prob2 group showed higher butyric acid concentrations at d 10 but lower BW at this age. During the early post-hatch period, the intestinal tract undergoes rapid structural and metabolic development, and butyrate may be preferentially used as an energy source for intestinal epithelial cells (Gaweł et al., 2025; Kinstler et al., 2024) and for mucin production (Liu et al., 2021) rather than directly supporting growth. As the gut microbiota stabilizes with age (Zhou et al., 2021), the influence of early microbial modulation may decrease, and compensatory growth may allow birds to reach similar BW at later stages.

Overall, the results of this study indicate that in-ovo probiotic administration does not act as a classical growth promoter under standard rearing conditions but can influence early physiological development, muscle characteristics, oxidative status, and microbial fermentation profiles. The absence of major differences in growth performance among treatments suggests that under controlled experimental conditions, both antibiotic growth promoters and probiotics may have limited effects on performance. However, the observed changes in BMY, oxidative status, and early cecal fermentation profiles indicate that early microbial intervention can influence host physiology beyond growth performance alone. These findings support the concept that in-ovo probiotic administration may act primarily through early-life programming of intestinal development, metabolism, and microbiota function rather than through direct growth stimulation. Therefore, in-ovo probiotics may represent a complementary strategy for antibiotic-reduced broiler production systems, particularly under commercial conditions where birds are exposed to environmental, nutritional, or microbial challenges

### Limitations

In this study, birds were raised under controlled experimental conditions with minimal environmental and pathogenic challenges, which may have limited the observable benefits of both antibiotic and probiotic treatments on growth performance. Under commercial production conditions or disease challenge models, differences among treatments may be more pronounced. Moreover, microbiota composition was not directly measured in this study, and conclusions regarding microbial modulation were inferred from cecal fermentation profiles. Future studies including microbiota sequencing and immune or intestinal development measurements would help clarify the mechanisms underlying the physiological effects observed. Finally, only one probiotic dose was evaluated during the animal phase following the screening trial, and dose-response effects during the grow-out period remain to be investigated.

## Conclusions

In-ovo administration of probiotic strains at doses up to 1.5 × 10⁷ CFU/egg did not adversely affect hatchability or chick quality, demonstrating good embryonic tolerance. Under the controlled conditions of this study, neither in-feed antibiotics nor in-ovo probiotics improved growth performance or feed efficiency relative to the negative control group. However, probiotic treatments influenced physiological and microbial-related parameters, including oxidative status and early cecal fermentation. These findings indicate that neither in-ovo probiotics nor in-feed antibiotics act as classical growth promoters under low-stress conditions but in-ovo probiotics may support bird health and resilience. Further studies conducted under commercial production conditions are warranted to validate these effects in environments where birds are exposed to greater environmental and health challenges.

## Declaration of AI and AI-assisted technologies in the writing process

During the preparation of this work the authors used ChatGPT in order to improve readability and language. After using this tool, the authors reviewed and edited the content as needed and take full responsibility for the content of the publication.

## CRediT authorship contribution statement

**Victor Grospiron:** Writing – original draft, Visualization, Investigation, Formal analysis, Data curation. **Jean-Michel Allard Prus:** Writing – review & editing, Investigation, Conceptualization. **Antony T. Vincent:** Writing – review & editing, Supervision, Methodology, Funding acquisition, Conceptualization. **Nabeel Alnahhas:** Writing – review & editing, Supervision, Resources, Methodology, Funding acquisition, Data curation, Conceptualization.

## Disclosures

The authors declare the following financial interests/personal relationships which may be considered as potential competing interests: Nabeel Alnahhas reports financial support was provided by Quebec Ministry of Agriculture Fisheries and Food. Nabeel Alnahhas reports financial support was provided by Natural Sciences and Engineering Research Council of Canada. Jean-Michel Allard Prus reports a relationship with Scott Hatchery (Quebec, Canada) that includes: employment. If there are other authors, they declare that they have no known competing financial interests or personal relationships that could have appeared to influence the work reported in this paper.

## Appendix Supplementary materials

Image, application 1

## Footnotes

Supplementary material associated with this article can be found, in the online version, at doi:10.1016/j.psj.2026.107026.
