# Supplementary material for: The potential of in-ovo probiotics to replace antibiotic growth promoters in broiler diets: Effect on performance, meat quality, cecal fermentation and oxidative stress indicators
Source: Poult Sci. 2026 Apr 28;105(8):107026. doi: 10.1016/j.psj.2026.107026 (PMC13185928; doi:10.1016/j.psj.2026.107026)
Supplement: Supplementary file 1 [file mmc1.docx]

**Table S1.** Ingredient and formulated nutrient composition of the starter diet (d 1 – d 10)

| **Ingredient Name** | **Amount (kg)** |
| --- | --- |
| Corn | 424.11 |
| Soybean meal | 248.0 |
| Wheat | 150.0 |
| Soya trituro, 44% | 100.0 |
| Meat and bone meal | 50.0 |
| Animal fat | 7.0 |
| DL-Methionine, 99% | 4.34 |
| Lysine sulfate, 70% | 3.6 |
| Lime stone | 3.2 |
| Salt | 2.7 |
| Threonine, 98.5% | 1.86 |
| Sodium bicarbonate | 1.2 |
| Vit. E, 50,000UI | 1.0 |
| Trace minerals premix | 1.0 |
| Liquid choline, 75% | 0.61 |
| L-Valine, 97% | 0.52 |
| L-Isoleucine, 98.5% | 0.46 |
| Vitamin Premix | 0.30 |
| Phytase | 0.10 |
| **Nutrient** | **Amount** |
| Crude protein, % | 23.71 |
| EMAn, kcal/kg | 2972.80 |
| EDB^1^, Meq/kg | 254.0 |
| dLys, % | 1.31 |
| dMeth&Cys/dLys | 0.76 |
| dMET/dLys | 0.56 |
| dLeu/dLys | 1.19 |

^1^Electrolytic dietary balance

**Table S2.** Ingredient and formulated nutrient composition of the grower diet (d 11 – d 21)

| **Ingredient name** | **Amount (kg)** |
| --- | --- |
| Corn | 417.57 |
| Soybean meal | 238.0 |
| Wheat | 200.0 |
| Soya trituro, 44% | 75.0 |
| Meat and bone meal | 34.0 |
| Animal fat | 17.0 |
| DL-Methionine, 99% | 3.84 |
| Lysine sulfate, 70% | 3.0 |
| Salt | 2.8 |
| Lime stone | 2.6 |
| Threonine, 98.5% | 1.5 |
| Sodium bicarbonate | 1.4 |
| Trace minerals premix | 1.0 |
| Vit. E 50,000 UI | 0.7 |
| Liquid choline, 75% | 0.61 |
| L-Valine, 97% | 0.38 |
| L-Isoleucine, 98.5% | 0.30 |
| Vitamin premix | 0.20 |
| Phytase | 0.10 |
| **Nutrient** | **Amount** |
| Crude protein, % | 21.67 |
| EMAn, kcal/kg | 3049.4 |
| EDB^1^, Meq/kg | 238.0 |
| dLys, % | 1.17 |
| dMeth&Cys/dLys | 0.78 |
| dMeth/dLys | 0.56 |
| dLeu/dLys | 1.24 |

^1^Electrolytic dietary balance

**Table S3.** Ingredient and formulated nutrient composition of the finisher diet (d 22 – d 31)

| **Ingredient Name** | **Amount (kg)** |
| --- | --- |
| Corn | 473.63 |
| Wheat | 200.0 |
| Soybean meal | 168.0 |
| Soy trituro, 44% | 100.0 |
| Meat and bone meal | 23.0 |
| Animal fat | 14.0 |
| Lime stone | 4.0 |
| DL-Methionine, 99% | 3.66 |
| Lysine sulfate, 70% | 3.60 |
| Salt | 2.80 |
| Sodium bicarbonate | 1.60 |
| Threonine, 98.5% | 1.52 |
| Trace minerals premix | 0.90 |
| L-Isoleucine, 98.5% | 0.70 |
| Vit. E 50,000 UI | 0.60 |
| L-Arginine, 96.5% | 0.59 |
| L-Valine, 97% | 0.58 |
| Liquid choline, 75% | 0.54 |
| Vitamin premix | 0.18 |
| Phytase | 0.10 |
| **Nutrient** | **Amount** |
| Crude protein, % | 19.5 |
| EMAn, kcal/kg | 3099.4 |
| EDB^1^, Meq/kg | 217.0 |
| dLys, % | 1.08 |
| dMeth&Cys/dLys | 0.79 |
| dMeth/dLys | 0.57 |
| dLeu/dLys | 1.24 |

^1^Electrolytic dietary balance
